# Self‐Reported Severity of Hand Eczema: Important Outcome but How Can We Measure It?

**DOI:** 10.1111/cod.14811

**Published:** 2025-05-16

**Authors:** Annika Wilke, Marc Rocholl, Christoph Skudlik, Kathrin Nordheider

**Affiliations:** ^1^ Department of Dermatology, Environmental Medicine and Health Theory Institute for Health Research and Education, Osnabrück University Osnabrück Germany; ^2^ Institute for Interdisciplinary Dermatological Prevention and Rehabilitation (iDerm) at the Osnabrück University Osnabrück Germany; ^3^ Lower Saxony Institute of Occupational Dermatology (NIB) Osnabrück Germany

**Keywords:** contact dermatitis, hand eczema, rating scales, self‐assessment, self‐rating, self‐reporting, validity

In clinical studies, the assessment of hand eczema severity by dermatologists or other health care professionals with a valid and reliable method that is sensitive to change is the methodical gold standard. In dermatology, validated scores such as the Hand Eczema Severity Index (HECSI) or the Osnabrück Hand Eczema Severity Index (OHSI) [1, 2] are commonly used. Nevertheless, there are research conditions under which patients' self‐reporting is required, for example, when dermatological consultations are not feasible [3]. For this purpose, we looked at the literature and our own, partly unpublished data to gain a deeper insight into the criterion validity of self‐reporting instruments for hand eczema severity and the correlation between different methods of patients' self‐reporting and an external, validated clinical gold standard [1].

## 1 Methods and Results

We were able to distinguish between complex self‐reporting instruments with more than one item to assess disease severity (e.g., the photographic guide [4], the Patient‐Oriented Eczema Measure (POEM) adapted for hand eczema [5], the Hand Eczema Extent Score (HEES) [6]) and 1‐item global rating scales (e.g., visual analogue scales [4], verbal rating scales [7], numerical rating scales [7], Table 1). Large correlation coefficients were described for the recently published patient‐HECSI [8] which measures self‐reported hand eczema severity quite accurately. However, it appears to be comparatively complex to complete since the validation study describes an initial phase in which the patient‐HECSI is explained to the patients comprising written educational material, a seven‐minute introductory video and illustrations of the signs of different hand eczema severity grades [8]. Apart from the patient‐HECSI, the different correlation coefficients between the other self‐report instruments and the respective gold standard mostly ranged from 0.4 to 0.6 whereas this is to be interpreted depending on the selected coefficient (Table 1).

**TABLE 1:** Correlations between instruments to assess patients' self‐reported severity of hand eczema and a clinical gold standard obtained by dermatologists or other health care professionals.

Instrument	Clinical gold standard	Correlation	Sample
Complex instruments (> 1 item to assess the disease severity of hand eczema)			
HEES [6]	HEES [6]	ICC^a^ = 0.61 [6]	*N* = 62 patients with hand eczema, Sweden [6]
Patient‐HECSI [8]	HECSI [8]	*r* ^b^ = 0.756 [8] ICC^a^ = 0.844 [8]	*N* = 72 patients with hand eczema, Denmark [8]
Photographic guide [4]	Photographic guide [4]	rho^c^ = 0.82 [4] kappa = 0.61^d^ [4]	*N* = 51 patients with hand eczema, Denmark [4]
Photographic guide [4]	OHSI [2]	rho = 0.415^c^ (T1, baseline) and rho = 0.543^c^ (T2, 1‐year follow‐up after intervention) [7]	*N* = 73 (T1) and *N* = 68 (T2) metal workers with WRSD, Germany [7]
Photographic guide [4]	OHSI [2]	rho = 0.564^c^ (T1, baseline) rho = 0.522^c^ (T2, 8–12 weeks follow‐up after intervention) (unpublished data^e^)	*N* = 256 (T1) and *N* = 219 (T2) patients with WRSD, Germany
POEM (adapted for hand eczema) [5]	OHSI [2]	*r* = 0.517^b^ (unpublished data)	*N* = 370 patients with WRSD, Germany
Global rating scales (1 single item to assess the disease severity of hand eczema)			
Visual analogue scale (VAS) of 0–10 [4]	Photographic guide [4]	rho^c^ = 0.52 [4]	*N* = 51 patients with hand eczema, Denmark [4]
Verbal rating scale (5‐point‐scale: *not at all, mild, moderate, strong, very strong*) [7]	OHSI [2]	rho = 0.536^c^ (T1, baseline) and rho = 0.547^c^ (T2, 1‐year follow‐up after intervention) [7]	*N* = 77 (T1) and *N* = 74 (T2) metal workers with WRSD, Germany [7]
Verbal rating scale (*5*‐point‐scale: *not at all, mild, moderate, strong, very strong*) [7]	OHSI [2]	rho = 0.473^c^ (T1, baseline) rho = 0.513^c^ (T2, 8–12 weeks follow‐up after intervention) (unpublished data)	*N* = 265 (T1) and *N* = 224 (T2) patients with WRSD, Germany
Verbal rating scale (5‐point‐scale: *clear, almost clear, mild, moderate, severe*) [5]	OHSI [2]	rho = 0.526^c^ (unpublished data)	*N* = 360 patients with WRSD, Germany [5]
Numerical rating scale (German school grades) from 1 (*very good*) to 6 (*unsatisfactory*) [7]	OHSI [2]	*r* = 0.477^b^ (T1, baseline) and *r* = 0.533^b^ (T2, 1‐year follow‐up after intervention) [7]	*N* = 76 (T1) and *N* = 70 (T2) metal workers with WRSD, Germany [7]
Numerical rating scale (German school grades) from 1 (*very good*) to 6 (*unsatisfactory*) [7]	OHSI [2]	*r* = 0.396^b^ (T1, baseline) and *r* = 0.434^b^ (T2, 8–12 weeks follow‐up after intervention) (unpublished data)	*N* = 267 (T1) and *N* = 219 (T2) patients with WRSD, Germany
Numerical rating scale from 0 to 10 [7]	OHSI [2]	*r* = 0.487^b^ (T1, baseline) and *r* = 0.416^b^ (T2 1‐year follow‐up after intervention) [7]	*N* = 77 (T1) and *N* = 70 (T2) metal workers with WRSD, Germany [7]
Numerical rating scale from 0 to 10 [7]	OHSI [2]	*r* = 0.405^b^ (T1, baseline) and *r* = 0.412^b^ (T2, 8–12 weeks follow‐up after intervention) (unpublished data)	*N* = 264 (T1) and *N* = 212 (T2) patients with WRSD, Germany

Abbreviations: HEES, Hand Eczema Extent Score; OHSI, Osnabrück Hand Eczema Severity Index; POEM, Patient‐Oriented Eczema Measure; WRSD, work‐related skin diseases.

^a^ ICC: Interclass correlation.

^b^ Pearson's correlation coefficient.

^c^ Spearman's rho/Spearman's correlation.

^d^ Cohen's kappa.

^e^ Uncontrolled pre‐post observational study of patients who took part in an outpatient skin protection seminar.

## 2 Discussion and Conclusion

In our opinion, the current state of research does not allow any clear recommendation as to which method should be used for the patients' self‐assessment of hand eczema severity and the choice of an appropriate instrument depends on the context and the aim of the study. Most correlations might not be sufficient to justify individual therapy decisions but they might be sufficient, for example, for longitudinal observational studies in health care research, if hand eczema severity needs to be assessed in a (large) patient cohort at several points in time [3, 7].

Ideally, instruments to measure hand eczema severity over time should be valid, reliable, responsive and easy‐to‐use from the patients' point of view. There are not yet sufficient validation studies that have fully explored the properties of self‐reporting instruments (e.g., test–retest‐reliability, sensitivity to change) as well as other possible (dis) advantages (e.g., applicability in digital settings). Furthermore, it should be noted that validated but yet different gold standards (e.g., OHSI, HECSI, photographic guide) have been used in previous studies to examine the criterion validity of the self‐reporting instruments so that a consensus for a uniform gold standard would be desirable. In line with this, it should be emphasised that the different correlation coefficients shown in Table 1 are not directly comparable. Due to the narrow focus of this contact point, further details on the studies referred to in Table 1 (e.g., population, methods) and their limitations could not be taken further into account. Further, this contact point is limited to self‐reporting instruments that were validated against validated clinical scoring systems [1].

While a core outcome set (e.g., clinical signs, patient‐reported symptoms) and core outcome instruments (e.g., the EASI [Eczema Area and Severity Index], POEM) for clinical trials already exist for atopic dermatitis (Harmonized Outcome Measures for Eczema [HOME], www.homeforeczema.org), this is not yet the case for hand eczema. Therefore, and resulting from our observations, it is pivotal that the HECOS (Hand Eczema Core Outcome Set) Initiative is dedicated to this topic and that the consensus process for a core domain set for hand eczema trials is currently underway [9]. Furthermore, it remains to be seen in the medium and long term whether and which instruments or scales will become established for self‐reporting the disease severity of hand eczema. Due to a different clinical picture and course of disease, the established survey instruments for clinical signs and self‐reported severity will most likely differ between atopic dermatitis and hand eczema, whereby the latter can be of different aetiological origins (atopic, irritant, allergic and overlapping diagnoses) [5]. Finally, we would like to share an observation (data not shown): simultaneously collecting data of the ‘subjective’, self‐reported and the ‘objective’, dermatologically assessed severity of hand eczema allows the identification of ‘special cases’ that may be familiar to many from clinical practice. These cases are expressed by a high subjective self‐reported disease severity and a low rated disease severity by the dermatologist (and vice versa) at the same time. It seems highly interesting to identify these patients by quantitative data collection in order to gain more insights into the patients' perspective and perception of hand eczema severity, for example, by qualitative interviews.

In summary, there are methodological challenges in this field to be addressed by future research: An instrument for self‐reported disease severity must fit to the specific study conditions and meet the methodological requirements. For example, it is not yet clear how and whether the photographic guide [4] (presentation of four degrees of hand eczema severity with four photos each on a single, colour‐printed A4 page) can be successfully transferred to and used in digital form (e.g., on smartphones with a small display) with similar validation results. This will be, however, increasingly relevant when considering digitalisation and digital data collection in research. Further, the time needed to complete a questionnaire must be considered. Less time‐consuming procedures with fewer items are most likely to be preferred by patients' and may probably increase response rates. It may therefore be of advantage to choose a validated, easy‐to‐use instrument especially when other scales and questionnaires need to be administered at the same time.

## Author Contributions

**Annika Wilke:** conceptualization, investigation, writing – original draft, methodology, writing – review and editing, formal analysis, project administration, data curation, resources. **Marc Rocholl:** conceptualization, investigation, writing – review and editing, methodology, data curation. **Christoph Skudlik:** resources, supervision, writing – review and editing. **Kathrin Nordheider:** formal analysis, writing – review and editing, methodology, supervision.

## Conflicts of Interest

All authors are professionally involved in the education, counselling, or medical care of patients with work‐related skin diseases. The authors declare that they have no further competing interests.
